# Muscle biopsy in genomic era: real-world diagnostic and clinical implications over 10 years

**DOI:** 10.1007/s00415-026-13917-8

**Published:** 2026-06-09

**Authors:** Dario Zoppi, Rosario Russo, Virginia Boemia, Martina De Maria, Anita Marciano e Ortolano, Francesca Vallefuoco, Elvira D’Amico, Gianni Di Costanzo, Anna Russo, Teresa Somma, Maria Nolano, Rosa Iodice, Luigi Maria Cavallo, Fiore Manganelli, Lucia Ruggiero

**Affiliations:** 1 Department of Neurosciences, Reproductive and Odontostomatological Sciences, University Hospital “Federico II”, Naples, Italy; 2 Division of Neurosurgery, Department of Neurosciences, Reproductive and Odontostomatological Sciences, University of Naples “Federico II”, Naples, Italy

**Keywords:** Muscle biopsy, Next-generation sequencing, HyperCKemia, Diagnostic yield

## Abstract

**Background:**

The diagnostic role of muscle biopsy has evolved with the increasing availability of next-generation sequencing (NGS). However, real-world data on its clinical impact in contemporary neuromuscular practice remain limited.

**Objective:**

To evaluate the diagnostic yield of muscle biopsy in a 10 year consecutive cohort, assessing concordance across clinical suspicion, histopathological findings, and final clinical diagnosis, and exploring demographic predictors of biopsy outcome.

**Methods:**

A retrospective cohort study of all consecutive muscle biopsies performed at a single tertiary neuromuscular center (2015–2025). Concordance analysis was performed at two levels: clinical suspicion vs. biopsy conclusion, and biopsy conclusion vs. final diagnosis at the last follow-up. Sex- and age-related differences across biopsy categories were evaluated using chi-square and Kruskal–Wallis tests.

**Results:**

Among 401 consecutive biopsies (56.9% male; median age 49 years), overall concordance between clinical suspicion and biopsy conclusion was 42.3%. Concordance with final clinical diagnosis was substantially higher, reaching 94.0% for idiopathic inflammatory myopathies (IIMs) among evaluable cases. Non-specific myopathic findings and normal biopsies accounted for 36.0% of cases; however, patients with non-specific myopathic findings were significantly more likely to receive a conclusive diagnosis at follow-up than those with normal biopsies (43.8% vs. 23.4%; OR 2.54, 95% CI 1.23–5.26, *p* = 0.011). Sex distribution differed significantly across biopsy categories (*p* < 0.001), with IIMs showing marked female predominance (69.1%).

**Conclusions:**

Muscle biopsy retains high diagnostic value in contemporary neuromuscular practice when applied to clinically selected patients. Beyond establishing specific diagnoses, biopsy findings carry prognostic significance and provide demographic signatures that reinforce their clinical validity.

## Introduction

Muscle biopsy has historically represented a cornerstone in the diagnostic evaluation of neuromuscular disorders, providing morphological, immunohistochemical, and ultrastructural information that directly informs clinical management [1, 2]. Over the past two decades, however, the diagnostic landscape has been profoundly reshaped by the advent of next-generation sequencing (NGS) and the availability of disease-specific serological biomarkers, as myositis-specific antibodies (MSAs) [3, 4]. These advances have led some clinicians to question whether muscle biopsy should remain a first-line investigation or be reserved for selected clinical scenarios [5].

Several recent studies have examined the diagnostic yield of muscle biopsy in the current era. Kapoor et al. (2022) reported that biopsy yielded a definitive or contributory diagnosis in 85.5% of adult-onset myopathy cases [6]. Conversely, other series have documented non-diagnostic rates ranging from 20 to 40%, particularly in patients referred for isolated hyperCKemia or non-specific clinical presentations [7, 8]. A systematic review by Garibaldi et al. (2024) highlighted substantial heterogeneity in biopsy yield across centers, underscoring the need for standardized outcome reporting [9].

Despite growing literature on biopsy yield, several questions remain underexplored. First, most studies have focused on specific disease subgroups (e.g., idiopathic inflammatory myopathies) rather than unselected consecutive cohorts [10, 11]. Second, concordance analysis between clinical suspicion, histopathological findings, and final longitudinal diagnosis is rarely reported [12]. Third, the clinical meaning of non-diagnostic biopsies—whether non-specific myopathic findings and normal morphology carry different prognostic implications—has not been systematically addressed. Finally, demographic variables, such as sex and age, have seldom been analyzed as correlates of biopsy outcome categories.

In this study, we present a comprehensive analysis of 401 consecutive muscle biopsies performed over a 10-year period at a single Italian tertiary neuromuscular center. Our objectives were: (1) to quantify diagnostic concordance at two levels (clinical suspicion → biopsy conclusion, and biopsy conclusion → final diagnosis); (2) to compare prognostic trajectories of non-specific myopathic findings versus normal biopsies; and (3) to explore demographic correlates of biopsy outcome categories.

## Materials and methods

### Study design and data source

This was a single-center, retrospective, observational cohort study. Data were extracted from an institutional clinical registry including all consecutive neuromuscular muscle biopsies performed at the University Hospital “Federico II” (Naples, Italy) between January 2015 and December 2025. For each case, we recorded: year of biopsy, referring unit, sex, age at biopsy, biopsy site, pre-biopsy clinical suspicion, histopathological conclusion, and conclusive diagnosis at last follow-up, defined as the diagnosis established at the most recent available follow-up visit by integrating all available clinical, electrophysiological, histopathological, and genetic data. Free-text clinical variables were harmonized into standardized diagnostic categories using predefined classification rules.

Genetic testing was performed by next-generation sequencing (NGS) using targeted myopathy gene panels, which were progressively expanded over the study period. It should be noted that several conditions were routinely diagnosed through specific genetic testing without requiring muscle biopsy, and were therefore not represented in this cohort: myotonic dystrophies (myotonic dystrophy type 1 (DM1) and myotonic dystrophy type 2), non-dystrophic myotonias, oculopharyngeal muscular dystrophy, and facioscapulohumeral dystrophy (FSHD). Similarly, dystrophinopathies in males were screened by multiplex ligation-dependent probe amplification (MLPA) and Pompe disease was excluded by acid alpha-glucosidase assay on dried blood spot (DBS) prior to biopsy referral.

### Muscle biopsy and histopathological analysis

In all cases, muscle biopsies were performed using an open procedure. In adult patients, the non-dominant biceps brachii was chosen as the standard muscle in the vast majority of cases. This choice allowed for standardization of results across patients, while also offering practical advantages during the surgical procedure and minimizing post-procedural discomfort. Only in selected cases where the clinical picture warranted it—particularly in the presence of focal manifestations—were specific muscles targeted instead. In pediatric patients, the quadriceps femoris was most frequently selected. Tissue samples were processed via flash-freezing in isopentane (methylbutane) cooled in liquid nitrogen. Subsequently, the samples were cut into 10-μm-thick cryostat sections and subjected to the following histological stains: Hematoxylin and Eosin (H&E), Gomori’s Trichrome (GT), Sudan Black, and Periodic Acid–Schiff (PAS). The following histoenzymatic activities were also evaluated: Nicotinamide Adenine Dinucleotide (NADH), Cytochrome-C-Oxidase (COX), Succinate Dehydrogenase (SDH), combined COX/SDH, Adenosine Triphosphatase (ATPase) at pH 9.4, 4.6, and 4.3, and Myophosphorylase (MFO). In selected cases, the analysis was further extended using immunohistochemical techniques with labeled antibodies directed against the following proteins: dystrophin (COOH-terminal, mid-rod, and NH-terminal domains), alpha-, beta-, gamma-, and delta-sarcoglycan, dysferlin, caveolin, spectrin, merosin, and laminin, as well as the lymphocytic markers CD20, CD4, CD8, and MAC. Histopathological diagnosis was conducted via light microscopy, while fluorescence microscopy was employed for immunohistochemical investigations. Samples showing alterations in oxidative metabolism or presenting with features consistent with mitochondrial or metabolic disorders were referred to an external laboratory for biochemical assays of mitochondrial respiratory chain enzyme activity and concurrent mitochondrial DNA (mtDNA) genetic analysis.

### Diagnostic categories and concordance definitions

Pre-biopsy clinical suspicion was classified as idiopathic inflammatory myopathies (IIMs; including dermatomyositis, inclusion body myositis, immune-mediated necrotizing myopathy [IMNM], anti-synthetase syndrome, and overlap myositis), mitochondrial disease, muscular dystrophy, congenital myopathy, metabolic myopathy, neuropathy/SMA, distal myopathy, non-specific myopathy, hyperCKemia (defined as CK levels exceeding 1.5 times the upper limit of normal [13]) with normal neurological examination, endocrine-related myopathy, and other/undetermined.

Histopathological conclusions were grouped as inflammatory myopathies (inflammatory infiltrates, perifascicular atrophy, rimmed vacuoles, MHC I sarcolemmal upregulation, necrosis/regeneration with paucity of inflammation), mitochondrial myopathy (COX-negative fibers, ragged red fibers, ragged blue fibers, COX-negative/SDH-positive fibers on combined staining), dystrophic changes (fiber size variation, necrosis, regeneration, endomysial fibrosis, fatty replacement, with abnormal immunohistochemistry for dystrophin, sarcoglycans, merosin, or α-dystroglycan), congenital myopathy (cores, nemaline rods, central nuclei, fiber type disproportion with type 1 fiber predominance), metabolic myopathy (vacuolar changes with acid phosphatase positivity, glycogen accumulation on PAS, or lipid storage on Sudan black), neurogenic changes (grouped atrophy, fiber type grouping, target/targetoid fibers, angular atrophic fibers), non-specific myopathic findings (myopathic changes without a pattern diagnostic of a specific entity), and normal biopsy. Concordance between clinical suspicion and biopsy findings was assessed using direct category matching with predefined equivalences: e.g., muscular dystrophy ≡ dystrophic changes; neuropathy/SMA ≡ neurogenic changes.

Biopsy outputs were additionally classified as informative (any specific diagnostic category or normal morphology) versus non-informative (non-specific myopathic findings). Normal morphology was considered informative, as it allowed for the exclusion of overt structural myopathies and helped redirect the diagnostic workup, although it should be interpreted with caution given that biopsy findings can be influenced by site selection, and some genetic neuromuscular disorders may present with normal or only non-specific myopathic alterations.

### Statistical analysis

The associations between categorical variables were evaluated using chi-square test, with Cramer’s V to estimate effect size. Post hoc analysis was conducted using standardized residuals (significance thresholds: |*r*|> 1.96 for *p* < 0.05; |*r*|> 2.58 for *p* < 0.01; |*r*|> 3.29 for *p* < 0.001). Sex distribution across biopsy categories was compared using chi-square. Age differences across categories were assessed using Kruskal–Wallis test. For the comparison between non-specific myopathic findings and normal biopsies, a 2 × 2 chi-square test was applied after dichotomizing follow-up outcome (conclusive diagnosis vs. no conclusive diagnosis). Post hoc pairwise comparisons were performed using Mann–Whitney U tests with Bonferroni correction for multiple comparisons. Odds ratios with 95% confidence intervals were calculated using the log method. Statistical significance was defined as *p* < 0.05 for all analyses. Analysis were performed using Python (version 3.10).

## Results

### Cohort characteristics

Between January 2015 and December 2025, 401 consecutive muscle biopsies were performed. Most biopsies originated from the Day Surgery Neurophysiopathology service (61.1%), followed by Neurology inpatient wards (19.5%) and external referrals (19.5%; *n* = 78). The cohort showed a male predominance (228 males, 56.9%; 173 females, 43.1%). Age data were available for 392 patients. Mean age was 44.1 ± 22.5 years, median 49 years (IQR 27–61), range 0.02–86 years. The biceps brachii was the most commonly sampled muscle (80.0%), followed by the quadriceps (18.0%). The demographic and clinical characteristics of the cohort are summarized in Table 1.

**Table 1:** Demographic and clinical characteristics of the study cohort (*N* = 401)

Variable	*N* (%)	Details
Sex		
Male	228 (56.9%)	
Female	173 (43.1%)	
Age (*N* = 392)		Mean 44.1 ± 22.5
Median (IQR)		49 (27–61)
Range		0.02–86 years
Biopsy site		
Biceps brachii	319 (80.0%)	
Quadriceps	74 (18.0%)	
Other	8 (2.0%)	
Referring unit		
Day Surgery NFP	244 (61.1%)	
Neurology inpatient	78 (19.5%)	
External referral	78 (19.5%)	

### Clinical suspicion and histopathological findings

The most frequent pre-biopsy suspicion was IIM (19.0%, *n* = 76), followed by mitochondrial disease (14.8%, *n* = 59), non-specific myopathy (14.5%, *n* = 58), and muscular dystrophy (11.0%, *n* = 44). In 13.0% of patients (*n* = 52), muscle biopsy was performed due to asymptomatic hyperCKemia. Histopathological conclusions included non-specific myopathic findings (20.0%, *n* = 80), inflammatory myopathies (16.8%, *n* = 67), normal morphology (16.0%, *n* = 64), mitochondrial myopathy (12.2%, *n* = 49), congenital myopathy (10.0%, *n* = 40), dystrophic changes (8.0%, *n* = 32), neurogenic changes (6.2%, *n* = 25), metabolic myopathy (4.2%, *n* = 17), and vacuolar myopathy (3.5%, *n* = 14).

### Sex and age distribution by biopsy category

**Fig. 1:**
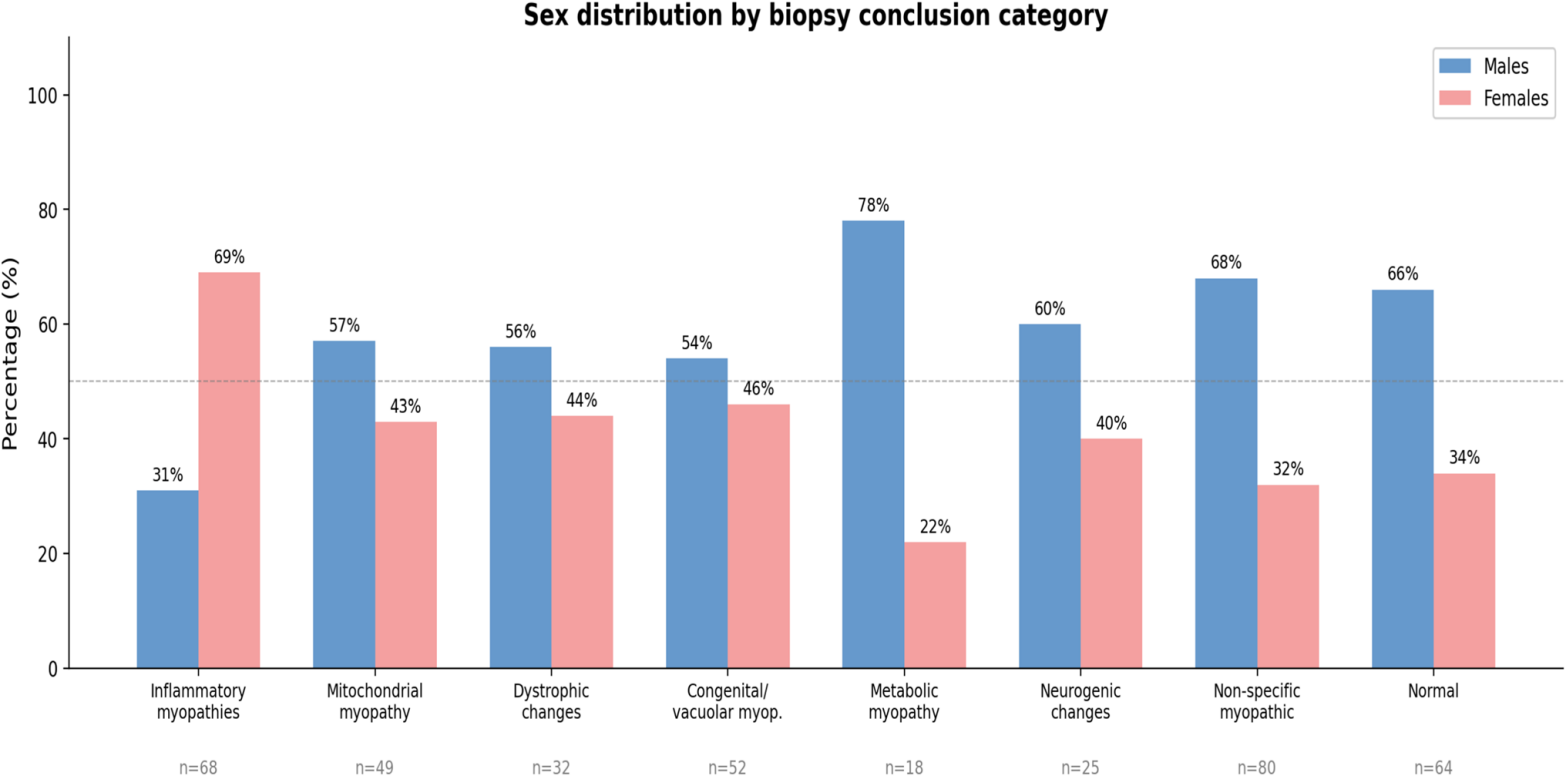
Sex distribution by biopsy conclusion category. Grouped bar chart showing the percentage of males (blue) and females (red) within each histopathological conclusion category. Inflammatory myopathies are the only category with marked female predominance (69.1%). *χ*^2^ = 27.91, df = 7, *V* = 0.267, *p* < 0.001

Sex distribution differed significantly across biopsy conclusion categories (*p* < 0.001; Fig. 1). IIMs were the only category with marked female predominance (69.1% F vs. 30.9% M, *n* = 68), consistent with the known epidemiology of dermatomyositis, IMNM, and anti-synthetase syndrome. Metabolic myopathy showed the highest male proportion (77.8%, *n* = 18), followed by non-specific myopathic findings (67.5%, *n* = 80) and normal biopsy (65.6%, *n* = 64). The latter two categories likely reflect the contribution of asymptomatic hyperCKemia, which is more prevalent in young males (Table 2).

**Table 2:** Sex and age distribution by biopsy conclusion category

Category	*N*	*M* (%)	*F* (%)	Med. age	IQR	Residual
IIMs	68	31%	69%	56	50–69	F***
Mitochondrial	49	57%	43%	52	32–64	ns
Dystrophic changes	32	56%	44%	52	44–66	ns
Congen./vacuolar	52	54%	46%	49	38–61	ns
Metabolic	18	78%	22%	42	34–55	M*
Neurogenic	25	60%	40%	52	40–60	ns
Non-spec. myop. findings	80	68%	33%	44	30–54	M**
Normal	64	66%	34%	41	27–55	M*

*χ*^2^ = 27.91, df = 7, *V* = 0.267, *p* < 0.001 (sex); KW H = 21.67, df = 7, *p* < 0.001 (age). Adjusted standardized residuals: * |r| > 1.96 (*p* < 0.05); ** |r| > 2.58 (*p* < 0.01); *** |r| > 3.29 (*p* < 0.001). M = male predominance; F = female predominance; ns = not significant.

**Fig. 2:**
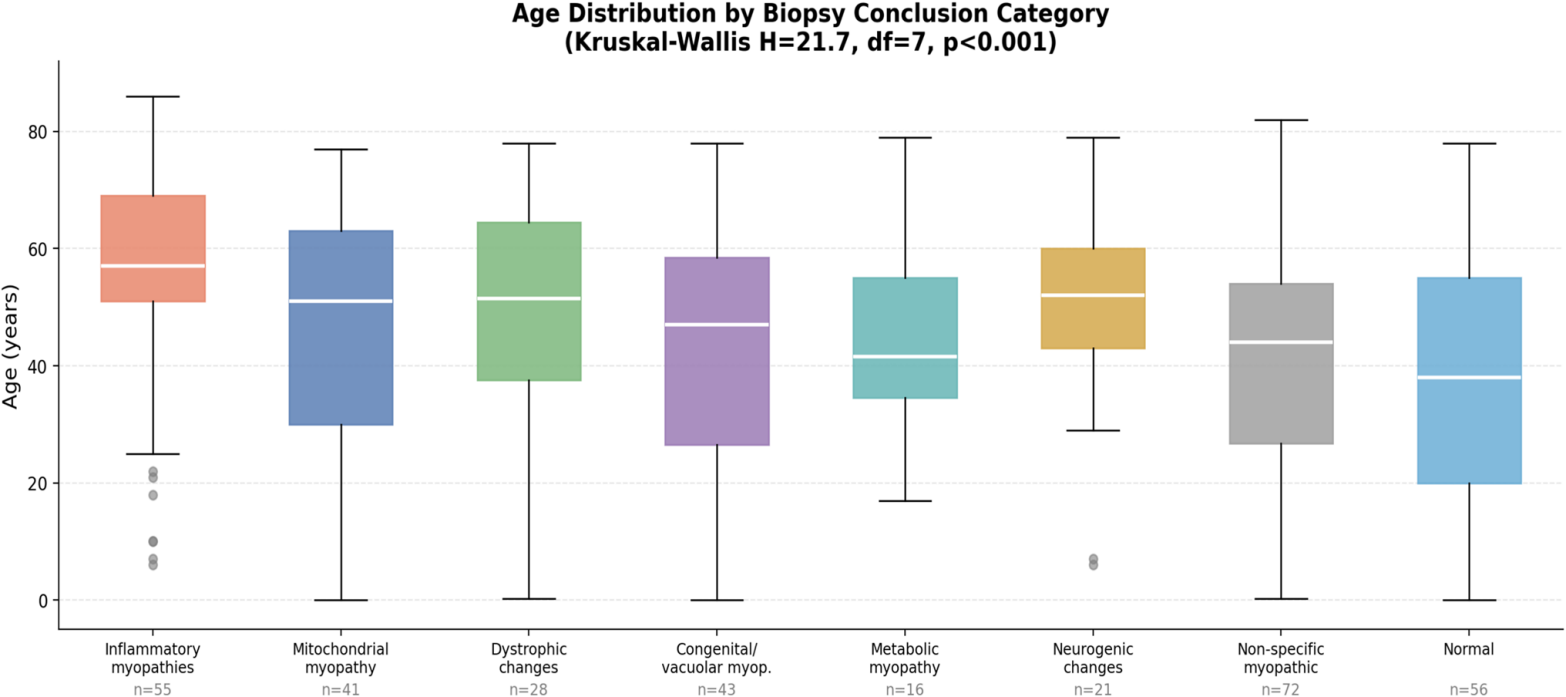
Age distribution by biopsy conclusion category (boxplot). Inflammatory myopathies show the highest median age (56 years), while non-specific myopathic findings and normal biopsies show younger profiles. Kruskal–Wallis H = 21.67, df = 7, *p* < 0.001

Age at biopsy varied significantly across categories (*p* < 0.001; Fig. 2). IIMs had the highest median age (56 years, IQR 50–69), while non-specific myopathic findings (median 44 years) and normal biopsy (median 41 years) showed younger age profiles, further supporting the hyperCKemia hypothesis in these groups.

### Concordance: clinical suspicion vs. biopsy conclusion

**Fig. 3:**
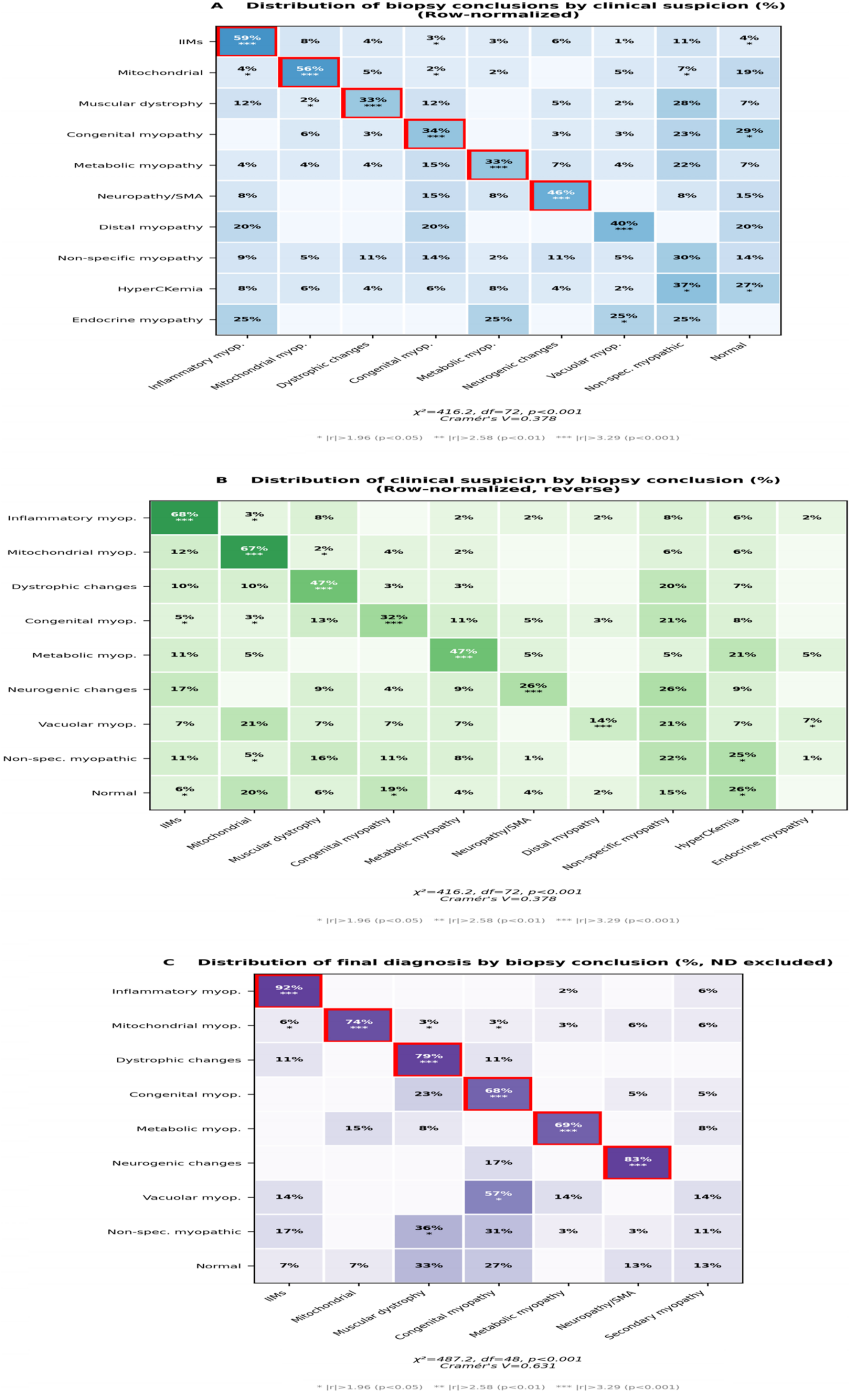
Concordance heatmaps. **A** Distribution of biopsy conclusions by clinical suspicion category (row-normalized %). **B** Reverse analysis: distribution of clinical suspicion categories within each biopsy conclusion. **C** Concordance between biopsy conclusion and final clinical diagnosis (excluding lost to follow-up). Colored borders indicate concordant cells; significance markers from standardized residual analysis (*p < 0.05,**p < 0.01, ***p < 0.001)

Among 310 cases with a predefined one-to-one category mapping, overall concordance between clinical suspicion and biopsy conclusion was 42.3% (131/310). Concordance was highest for IIMs (55.3%, 42/76) and mitochondrial disease (54.2%, 32/59), and lowest for metabolic myopathy (30.8%, 8/26), muscular dystrophy (31.8%, 14/44), and congenital myopathy (35.3%, 12/34). The overall association was highly significant (*p* < 0.001). Standardized residual analysis confirmed the strongest positive associations along the diagonal (Fig. 3A). Reverse analysis—distribution of clinical suspicion categories within each biopsy conclusion—revealed that IIMs (62.7%) and mitochondrial myopathy (65.3%) had the highest proportion of concordant referrals, whereas non-specific myopathic findings originated from a heterogeneous clinical spectrum (Fig. 3B).

### Final diagnosis and biopsy-to-diagnosis concordance

A conclusive diagnosis was documented in 223/401 cases (55.6%). In the remaining cases, longitudinal diagnostic documentation was unavailable, consistent with the referral dynamics of a tertiary center where patients are frequently repatriated to referring institutions after biopsy reporting. In evaluable cases, agreement between biopsy conclusion and final diagnosis was highest for IIMs (47/50, 94.0%), followed by dystrophic changes (15/19, 78.9%), metabolic myopathy (9/12, 75.0%), mitochondrial myopathy (26/35, 74.3%), and congenital/vacuolar myopathy (19/28, 67.9%). The overall association was strong (*p* < 0.001; Fig. 3C, Table 3).

**Table 3:** Concordance between biopsy conclusion and conclusive diagnosis at last follow-up

Biopsy conclusion	Total *N*	Evaluable *N*	Concordant	% (eval.)	% (total)
IIMs	67	50	47	94.0%	70.1%
Neurogenic changes	25	6	5	83.3%	20.0%
Dystrophic changes	32	19	15	78.9%	46.9%
Metabolic myopathy	17	12	9	75.0%	52.9%
Mitochondrial	49	35	26	74.3%	53.1%
Congen./vacuolar	54	28	19	67.9%	35.2%

*χ*^2^ = 649.1, df = 72, *V* = 0.527, *p* < 0.001. Evaluable = cases with available longitudinal diagnostic documentation

### Prognostic significance of non-diagnostic biopsies

**Fig. 4:**
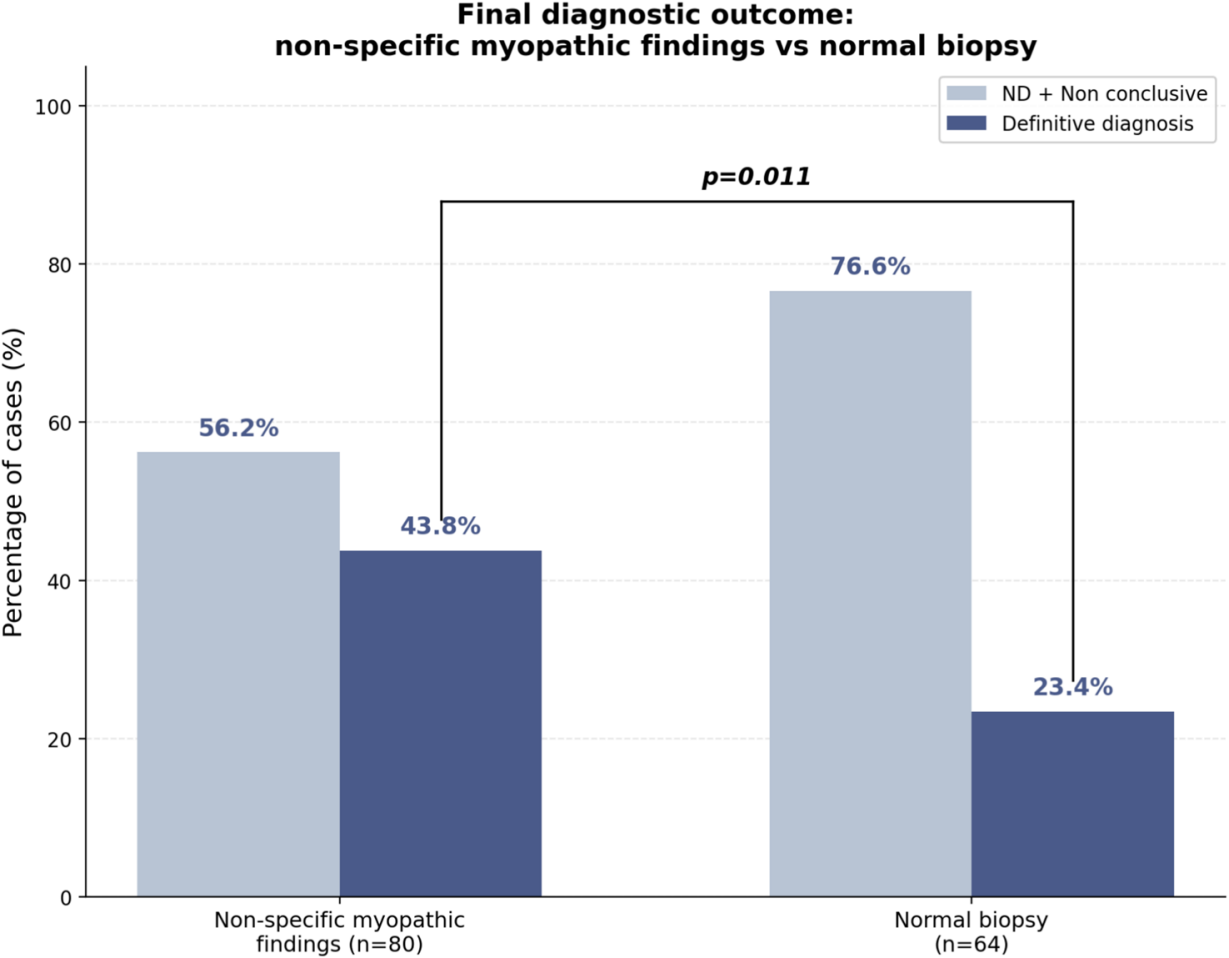
Diagnostic outcome comparison between non-specific myopathic findings (*n* = 80) and normal biopsy (*n* = 64). Patients with non-specific findings were significantly more likely to achieve a conclusive diagnosis during follow-up (*χ*^2^ = 6.47, *p* = 0.011; OR 2.54, 95% CI 1.23–5.26)

Patients with non-specific myopathic findings at biopsy (*n* = 80) were significantly more likely to achieve a conclusive diagnosis during follow-up compared with those with normal morphology (*n* = 64): 43.8% vs. 23.4% (*p* = 0.011; OR 2.54, 95% CI 1.23–5.26; Fig. 4). Among patients with non-specific myopathic findings who received a final diagnosis, the most common categories were muscular dystrophy (16.2%), congenital myopathy (13.8%), and IIMs (7.5%). In these cases, diagnosis was established via comprehensive genetic analysis, segregation studies, and muscle MRI, correlating with clinical findings. In the normal biopsy group, the proportion without diagnostic clarification was higher, with a substantial fraction of patients not returning for further evaluation, consistent with the benign nature of their underlying condition.

## Discussion

In this 10 year retrospective cohort of 401 consecutive muscle biopsies at a single Italian tertiary center, we found that muscle biopsy continues to provide clinically actionable information in a substantial proportion of cases, with category-dependent performance that aligns with and extends prior reports. Three principal findings emerge from our data.

### Diagnostic concordance in context

The overall concordance between biopsy conclusion and final clinical diagnosis was very high indicating that biopsy findings—when integrated with clinical, serological, and genetic data—contribute to accurate final diagnoses. The highest predictive accuracy was observed for IIMs (94.0% among evaluable cases), a finding with direct therapeutic implications, as these conditions are amenable to immunosuppressive treatment and prompt histological confirmation guides early and most appropriate intervention [14, 15]. Although in patients with positive myositis-specific antibodies, a compatible clinical picture, and muscle MRI findings indicative of active inflammation, a diagnosis of IIM may be established without biopsy, current ENMC and EULAR classification criteria still incorporate histopathological findings as supportive or confirmatory elements. This is particularly relevant in the context of seronegative IIMs, where biopsy remains the only reliable diagnostic tool [16, 17].

Notably, the concordance between clinical suspicion and biopsy conclusion (42.3%) may appear modest but should be interpreted in the context of the unselected, consecutive nature of our cohort, which includes a substantial proportion of diagnostically challenging presentations (hyperCKemia 13.0%, non-specific myopathy 14.5%). In more selected populations, higher concordance rates have been reported: Kapoor et al. found an 85.5% diagnostic yield when restricting analysis to patients with suspected myopathy and supportive EMG/MRI findings [6], and a recent multicenter study reported 92.2% concordance in a cohort where both clinical and biopsy diagnoses were pre-categorized [18]. Our figure therefore reflects the real-world diagnostic complexity inherent to an unselected consecutive cohort from a tertiary referral center. Importantly, the diagnostic value of muscle biopsy is substantially enhanced by appropriate pre-biopsy investigations aimed both at excluding alternative diagnoses—including blood tests, enzymatic screening, and targeted genetic testing in suggestive phenotypes—and at optimizing diagnostic yield through improved sampling selection, for example by muscle MRI. Even in diagnostically challenging cases, muscle biopsy maintains its prognostic value, at the very least by confirming or ruling out myopathic involvement.

### Clinical meaning of non-diagnostic biopsies

A distinctive contribution of our study is the systematic comparison between non-specific myopathic findings and normal biopsy as two distinct non-diagnostic pathways. Patients with non-specific histopathology were more than twice as likely to receive a conclusive diagnosis during follow-up (OR 2.54, *p* = 0.011), suggesting that subtle structural abnormalities—even when insufficient for a specific biopsy diagnosis—may reflect early, evolving, or atypical disease stages. Conversely, the high rate of diagnostic non-closure in the normal biopsy group is consistent with a population enriched for benign conditions (e.g., idiopathic hyperCKemia).

This distinction has practical implications: non-specific myopathic findings should prompt continued diagnostic workup, while a normal biopsy may carry a more reassuring prognosis for a subset of patients, particularly young males with isolated hyperCKemia [19]—consistent with the younger age and male predominance we observed in this category.

### Demographic signatures of biopsy categories

The significant association between sex and biopsy outcome category (*p* < 0.001) and between age and biopsy outcome (*p* < 0.001) adds a novel dimension to the interpretation of biopsy data. The striking female predominance in IIMs (69.1%) mirrors published epidemiological data for dermatomyositis and IMNM [15, 20], serving as an internal validation of our classification system. No significant age-related associations were found for neurogenic or mitochondrial forms after correction for multiple comparisons. The male predominance and younger age profile observed in non-specific myopathic findings and normal biopsies further support the hypothesis that these categories are enriched with hyperCKemia referrals—a condition known to disproportionately affect young males [19]. These findings suggest that demographic variables may assist in pre-test probability estimation when planning muscle biopsy.

### Muscle biopsy in the genomic era

Our data support a refined, complementary role for muscle biopsy alongside genetic testing rather than a competitive one. In our center, patients undergo a structured pre-biopsy workup that includes acid alpha-glucosidase assay on DBS to exclude Pompe disease and EMG to identify neurogenic conditions, which are generally diverted from biopsy. Moreover, several conditions are routinely diagnosed through specific genetic testing without requiring muscle biopsy, including myotonic dystrophy type 1 (DM1) and myotonic dystrophy type 2 (DM2/PROMM), non-dystrophic myotonias, oculopharyngeal muscular dystrophy, facioscapulohumeral dystrophy (FSHD), dystrophinopathies in males identified by MLPA and spinal muscular atrophy (SMA) through SMN1 deletion testing. Nonetheless, approximately 5% of patients in our cohort ultimately received one of these diagnoses—mostly FSHD—having been referred from external facilities without prior genetic screening. Only two cases of late-onset SMA type III were identified, while no cases of Kennedy disease were observed. This pre-selection means that biopsy in our cohort was predominantly performed in cases where genetic testing alone was insufficient or where histopathological characterization was needed to guide treatment. In the current era, NGS panels can resolve a substantial proportion of hereditary myopathies [3, 21]. In our cohort, the majority of patients underwent biopsy first and were subsequently tested with targeted NGS myopathy panels—which have been progressively expanded over the study period—and in some cases previously unresolved patients were retested on updated panels. However, the diagnostic paradigm is shifting: genetic testing increasingly precedes biopsy, and histopathology is more often used to validate variants of uncertain significance (VUS), where specific findings such as protein deficiency on immunohistochemistry or characteristic ultrastructural abnormalities can provide critical evidence for variant reclassification. Biopsy remains extremely useful in immune-mediated conditions requiring histological characterization for treatment decisions, particularly when antibody testing alone is insufficient to fully characterize the clinical picture, and in the differential diagnosis of ambiguous clinical presentations where histopathology may refine and redirect the diagnostic workup. [5, 17]. Muscle biopsy also provides invaluable material for functional studies, proteomics [22], and ultrastructural analysis that cannot be replicated by molecular approaches alone [23, 24]. The high concordance rates observed in our cohort for treatable conditions (IIMs, metabolic myopathies) underscore this complementary value. A detailed genotype–phenotype analysis, including variant classification and reclassification over time, was beyond the scope of this study but represents a natural extension of this work.

### Limitations

The main limitation of this study is the retrospective design and the single-center nature of the data. As is typical in tertiary referral settings, longitudinal diagnostic documentation was not available for all patients, as many were repatriated to referring centers after biopsy reporting; this limits the number of evaluable cases for biopsy-to-diagnosis concordance analyses but does not affect the concordance rates calculated among evaluable cases. Additional limitations include possible residual misclassification inherent to harmonizing free-text clinical variables.

## Conclusions

Muscle biopsy retains significant diagnostic and prognostic value in contemporary neuromuscular practice, although perhaps no longer as a first-line investigation. Its diagnostic yield appears highest when applied to appropriately selected patients with a targeted diagnostic suspicion and when pre-procedural investigations—including blood-based enzymatic and genetic testing—help guide the indication and biopsy site selection. Concordance with final clinical diagnosis is high for treatable conditions, particularly IIMs. Muscle biopsy also retains an important validation role for uncertain biochemical findings, such as isolated hyperCKemia, and for the interpretation of genetic variants of uncertain significance through immunohistochemistry and protein blot studies, as well as for long-term tissue storage enabling future reassessment in unresolved cases. Our analysis shows that non-specific myopathic findings and normal biopsies represent distinct prognostic trajectories, with different implications for a tailored follow-up. Both scenarios warrant careful consideration. Indeed, a normal biopsy does not definitively exclude a myopathy or underlying neuromuscular disorder. Nonetheless, in cases with abnormal—even if non-specific—biopsy findings, the final diagnostic rate remains approximately 2.5-fold greater than in patients with normal biopsy findings (OR = 2.54, *p* = 0.011) suggesting that this distinction carries clinical relevance and should be taken into account in the diagnostic reasoning, also to avoid the risk of overdiagnosis, particularly when genetic findings are of uncertain significance. Certain demographic variables were associated with specific biopsy outcome categories and may provide useful context when interpreting biopsy results. For instance, inflammatory myopathy should be given greater diagnostic consideration in female patients, while young males referred for isolated hyperCKemia are more likely to yield normal or non-specific biopsy findings. These findings support the continued integration of muscle biopsy into modern diagnostic algorithms alongside genetic testing and advanced biomarkers.

## Data Availability

The datasets used and/or analyzed during the current study are available from the corresponding author on reasonable request.
